# Arterial desaturation rate does not influence self‐selected knee extension force but alters ventilatory response to progressive hypoxia: A pilot study

**DOI:** 10.14814/phy2.15892

**Published:** 2024-01-03

**Authors:** Saro D. Farra, Ira Jacobs

**Affiliations:** ^1^ Faculty of Kinesiology & Physical Education University of Toronto Toronto Ontario Canada; ^2^ Tanenbaum Institute for Science in Sport, University of Toronto Toronto Ontario Canada

**Keywords:** afferent feedback, arterial oxygen saturation, hypoxia, rate of physiological strain development, sequential gas delivery

## Abstract

The absolute magnitude and rate of arterial desaturation each independently impair whole‐body aerobic exercise. This study examined potential mechanisms underlying the rate‐dependent relationship. Utilizing an exercise protocol involving unilateral, intermittent, isometric knee extensions (UIIKE), we provided sufficient reperfusion time between contractions to reduce the accumulation of intramuscular metabolic by‐products that typically stimulate muscle afferents. The objective was to create a milieu conducive to accentuating any influence of arterial desaturation rate on muscular fatigue. Eight participants completed four UIIKE sessions, performing one 3 s contraction every 30s at a perceived intensity of 50% MVC for 25 min. Participants voluntarily adjusted their force generation to maintain perceptual effort at 50% MVC without feedback. Reductions in inspired oxygen fraction (F_I_O_2_) decreased arterial saturation from >98% to 70% with varying rates in three trials: FAST (5.3 ± 1.3 min), MED (11.8 ± 2.7 min), and SLOW (19.9 ± 3.7 min). F_I_O_2_ remained at 0.21 during the control trial. Force generation and muscle activation remained at baseline levels throughout UIIKE trials, unaffected by the magnitude or rate of desaturation. Minute ventilation increased with hypoxia (*p* < 0.05), and faster desaturation rates magnified this response. These findings demonstrate that arterial desaturation magnitude and rate independently affect ventilation, but do not influence fatigue development during UIIKE.

## 1 INTRODUCTION

Whole body aerobic exercise performance deteriorates in hypoxia (Amann et al., 2006; Amann et al., 2007; Clark et al., 2007; Goodall et al., 2012) as a consequence of developing peripheral and central fatigue. While hypoxic (HYP) exercise is associated with reductions in motor unit recruitment (Kayser, 2003), there is debate surrounding the origin and nature of the neural mechanisms that regulate this impairment. Some suggest that muscle activation decreases in response to inadequate oxygen (O_2_) delivery and utilization by muscle (Amann et al., 2006) and cerebral (Amann et al., 2007; Millet et al., 2012; Rasmussen et al., 2010) tissues. Others propose that muscle activation is proactively reduced to prevent a critical decline in tissue oxygenation from developing in vital organs, thereby preserving the integrity of physiological systems (Noakes et al., 2001; Noakes & Marino, 2007). Despite our understanding of the neural mechanisms that influence motor unit recruitment during HYP exercise, most of the published research exploring the impact of hypoxia on fatigue has investigated how absolute changes in HYP stress/strain influence exercise performance. Much less is known about how the rate of change in HYP stress/strain impacts the neuromuscular system during exercise. Considering this “rate of change” aspect is important as it may provide valuable insights into unknown mechanisms governing human exercise performance.

To advance our understanding of hypoxia‐induced fatigue, our laboratory recently demonstrated that self‐selected cycling intensity was impaired with decreasing arterial oxygen saturation (S_P_O_2_) during exercise at a constant rating of perceived exertion (RPE) (Farra et al., 2017). Although this finding corroborates previous research (Amann et al., 2006; Amann et al., 2007; Clark et al., 2007; Goodall et al., 2012), the novel outcome of Farra et al. (2017) was that the magnitude of this impairment was amplified when progressive levels of HYP strain were continuously applied at a faster rate, at a time when O_2_ availability was similar across conditions (S_P_O_2_ = 80%). In addition, the magnified impairment of self‐selected cycling intensity in response to faster desaturation rates was accompanied by a greater reduction in muscle activation, suggesting that central processes were involved. It remains unknown whether the signal responsible for the modulation of self‐selected cycling intensity in response to faster desaturation rates originated within the CNS or peripheral tissues.

Elucidating the mechanisms that mediate the rate dependent relationship between arterial desaturation and exercise performance is difficult with continuous whole‐body exercise. The integrative physiological challenges associated with continuous whole body HYP exercise, such as convective O_2_ transport (Calbet & Lundby, 2009) and blood flow redistribution (Amann, Pegelow, et al., 2007) may simultaneously influence the cardiorespiratory system as well as central and peripheral neuromuscular processes. However, unilateral, intermittent, isometric knee extension is an exercise model that engages a much smaller muscle mass, for which systemic O_2_ delivery is not limiting even during maximal intensity exercise (Andersen & Saltin, 1985). The utility of this mode of exercise is related to the fact that when O_2_ transport is impaired, the O_2_ requirement of the working muscle can be met by increasing blood flow to the exercising limb (Rowell et al., 1986). In addition, the rest periods between intermittent isometric contractions facilitate muscle reperfusion, enhancing O_2_ delivery and metabolite washout. Consequently, intramuscular metabolic disturbances would be attenuated, reducing the accumulation of metabolites that impair muscular contractile processes (Allen et al., 2008) and stimulate peripheral muscle afferents (Rotto & Kaufman, 1988). It has been suggested that elevated feedback from peripheral muscle afferents mediate an increased RPE and decreased endurance time to task failure at a given exercise intensity during knee extension exercise (Amann et al., 2013). Therefore, unilateral, intermittent, isometric knee extension may be a useful model to further our understanding about the mechanisms that influence the rate dependent relationship between arterial desaturation and voluntary muscle force production.

Compared to other studies that have used more strenuous intermittent unilateral isometric contraction protocols (Fulco et al., 1994; Fulco et al., 2001; Katayama et al., 2007; Katayama et al., 2010; Rupp et al., 2015), we deliberately selected an exercise model that did not challenge the capacity for O_2_ delivery and utilization. Our protocol involved repeated 3 s static contractions at a perceived intensity of 50% maximal voluntary contraction (MVC) followed by 27 s of rest. Such a protocol should minimize changes in the intramuscular milieu normally associated with increases in metabolic rate as well as the associated inhibitory sensory feedback originating from peripheral muscle afferents. This would allow an investigation of the direct and independent influence of desaturation rate on the anatomical structures within the CNS that are responsive to hypoxia and play a role in controlling muscle activation. Using an exercise protocol that is based on perceptual effort, participants were free to adjust their voluntary force production to maintain the perceived target intensity in response to the HYP manipulations. Cooper et al. (1979) demonstrated that participants can reliably reproduce voluntary force as a percentage of their MVC in the absence of any knowledge about the exercise intensity for a single contraction, while the current study demonstrates that this consistency is maintained over the duration of an exercise trial. Such a consistent response lends itself to experimental manipulation seeking to separate the impact of the absolute magnitude of HYP strain from the rate of change in HYP strain on self‐selected force production.

The aim of this study was to investigate how different rates of arterial desaturation impact self‐selected force production during unilateral, intermittent, isometric knee extension exercise, while maintaining a consistent level of perceived effort. We hypothesized that if muscle activation is directly influenced by the rate of arterial desaturation, then impairments to self‐selected force generation may be more pronounced with faster rates of HYP strain development, even when potential intramuscular metabolic disturbances are mitigated. And, in contrast, should participants maintain a consistent self‐selected level of force production throughout the HYP trials, it would lend support to the proposition that feedback from peripheral muscle afferents contributes to the rate dependent relationship between arterial desaturation and muscle activation.

## 2 MATERIALS AND METHODS

### 2.1 Participants

Ten participants volunteered to participate in this study. Participants were healthy individuals with at least 1 year of resistance training experience. The data of two participants were excluded from the analysis as one voluntarily withdrew prior to completing all study requirements, and another was unable to consistently achieve an S_P_O_2_ of 70% across all HYP trials. The data of five males (30 ± 8 years, 178 ± 8 cm, 82 ± 13 kg) and three females (28 ± 5 years, 160 ± 9 cm, 61 ± 7 kg) were subsequently analyzed. Based on a within‐subject standard deviation of 5% MVC force (determined from the control trial) in the main outcome variable, self‐selected force generation at a perceived intensity of 50% MVC, a sample size of eight participants would detect an 8% MVC force difference between conditions at a two‐sided 0.05 level, with a power of 0.80. Power calculations were carried out using online software (http://hedwig.mgh.harvard.edu). Participants were informed of the risks associated with the study and a written informed consent was obtained. Participants were asked to avoid ingesting any food or drink (besides water) for 2 h before testing, to refrain from consuming caffeinated food or beverages for 12 h before testing, and to not engage in strenuous exercise for 24 h before any testing session.

### 2.2 Protocol

Participants reported to the laboratory on five occasions, with sessions separated by at least 48 h. During the first visit, basic physical characteristics were measured, participants were familiarized with the testing procedures, and the relationship between the partial pressure of inspired O_2_ (P_I_O_2_) and S_P_O_2_ was determined as described below.

**FIGURE 1:**
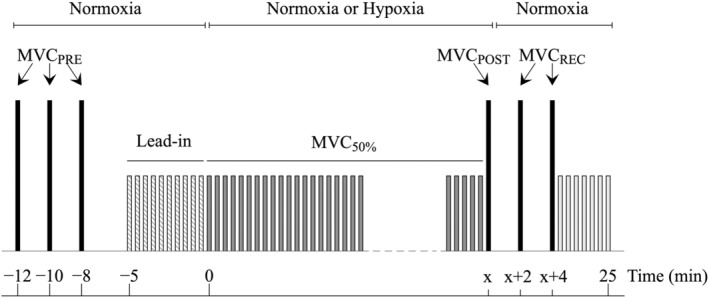
Schematic view of the experimental protocol.

In the four subsequent visits, the study utilized a single‐blind, crossover design with HYP and control (CON) trials completed in a counterbalanced order. These trials occurred at the same time of day and under consistent laboratory conditions. Participants were seated in a dynamometer designed for unilateral, isometric knee extension of the right leg. The protocol commenced with participants performing three 5 s MVCs separated by 2 min. The trial with the highest force output was designated as MVC_PRE_ and served as the reference for participants when selecting their level of force generation during the exercise trial. Following the third MVC, participants started a 5 min lead‐in period where they performed one 3 s contraction every 30 s while inhaling gas with a fraction of inspired O_2_ (F_I_O_2_) of 0.21. The lead‐in period allowed participants to establish a level of voluntary effort that they perceived to correspond to 50% of MVC_PRE_. Immediately after the lead‐in period, participants began the exercise trial (MVC_50%_), during which they continued to perform one 3 s contraction every 30 s at an intensity they perceived as 50% of MVC_PRE_. Participants received no visual or verbal cues regarding their actual force generation, except for occasional reminders to adjust force output as they fatigued to maintain their level of perceived effort at the target level. On three of these days, F_I_O_2_ was continuously reduced to desaturate arterial blood from baseline (> 98%) to 70% while maintaining isocapnia. The desaturation protocol began at the onset of MVC_50%_ and progressed approximately linearly over three different time periods: 5 min, 15, min, and 25 min for the FAST, MED, and SLOW conditions respectively. During CON, participants continued to breath gas with a F_I_O_2_ of 0.21. The MVC_50%_ trial ended when S_P_O_2_ reached 70% in the HYP trials or when 25 min had elapsed during CON. 30 s after the final contraction of MVC_50%_, participants performed a single 5 s MVC (MVC_POST_). Immediately following MVC_POST_, F_I_O_2_ was surreptitiously switched back to 0.21 for the remainder of the 25 min trial, leading to the restoration of S_P_O_2_ to baseline levels in approximately 90 s. Two minutes following MVC_POST_, participants proceeded to perform two additional 5 s MVCs with a 2 min recovery between them. The trial in which the highest force was recorded was labeled as MVC_REC_. Thirty seconds after the final MVC attempt, participants resumed performing one 3 s contraction every 30 s at a perceived intensity of 50% MVC_PRE_ until the end of the 25 min trial. A visual schematic of the protocol can be found in Figure 1.

While participants were informed about the absolute level of arterial desaturation they would experience, they were unaware of the F_I_O_2_ during the exercise trials. Moreover, participants were kept naive about the hypotheses of the study as they were not informed of the varying desaturation rates among trials. To maintain participant blinding, they were asked to complete the full 25 min trial, irrespective of the time required to reach an S_P_O_2_ of 70%. The experimental trials were terminated either when the participant indicated their desire to stop or when the investigator observed symptoms indicative of severe HYP strain. All participants were informed of the termination criteria.

### 2.3 Hypoxia administration

#### 2.3.1 Isocapnic hypoxia system

The isocapnic hypoxia system (IHS) that was developed in our laboratory (Farra et al., 2016) was used in this investigation to decrease S_P_O_2_ while clamping the partial pressure of end‐tidal carbon dioxide (PET_CO2_) within 2 mmHg of starting values throughout and among all exercise trials (Table 1). Isocapnia was maintained utilizing the principles of sequential gas delivery (Slessarev et al., 2007; Sommer et al., 1998; Somogyi et al., 2005). Briefly, the IHS included a 3‐way breathing manifold (Model # 100505, Thornhill Research, Canada) connected to an inspiratory and expiratory reservoir. The configuration of the manifold was such that it first delivered fresh gas from the inspiratory reservoir, and when this source of gas was depleted, it sequentially delivered previously expired gas from the expiratory reservoir. The flow of fresh gas into the inspiratory reservoir, as well as its composition, were controlled by custom computer software (LabView, National Instruments, Austin) regulating two independent mass flow controllers (Model # 32907–77, Cole‐Parmer, Montreal), with each mass flow controller adjusting the flow rate of one source gas (Tank 1–21% O_2_, balance N_2_; Tank 2–5% O_2_, balance N_2_). At the start of the lead‐in period and throughout the MVC_50%_ trials, the investigators adjusted the gas flow rate into the inspiratory reservoir to clamp PET_CO2_, and the software automatically regulated changes in gas composition according to user‐defined equations (see below).

**TABLE 1:** PET_CO2_ (mmHg) during MVC_50%_. During CON, PET_CO2_ was calculated at the beginning, one‐third, two‐thirds, and at the end of the time required to complete the trial. These values were compared to the data collected at the defined S_P_O_2_ values of 100%, 90%, 80%, and 70% during each HYP trial respectively.

	FAST	MED	SLOW	CON
~ 100%	37 ± 4	36 ± 3	37 ± 5	35 ± 4
90%	37 ± 4	36 ± 3	37 ± 5	35 ± 4
80%	36 ± 5	37 ± 4	37 ± 5	35 ± 3
70%	36 ± 5	35 ± 4	35 ± 6	35 ± 4

*Note*: Data are mean ± SD (*n* = 8). Data analyzed using a two‐way repeated measures ANOVA. No statistical differences were found.

Abbreviation: PET_CO2_, partial pressure of end‐tidal carbon dioxide.

#### 2.3.2 Modeling the P_I_O_2_‐S_P_O_2_ relationship

While sitting in the dynamometer, participants were connected to the IHS while F_I_O_2_ levels were decreased linearly over a 15 min period from 0.21 to approximately 0.08. F_I_O_2_ was multiplied by barometric pressure to calculate P_I_O_2_. S_P_O_2_ was continuously recorded throughout the desaturation procedure. The relationship between P_I_O_2_ and S_P_O_2_ was modeled with a second order polynomial function. Equation #1 exemplifies this function for one subject:

(1) $$P_{I}O_{2}=0.04x^{2}-4.5x+194.2$$

where

$$x=S_{P}O_{2}\;\left(\%\right).$$

#### 2.3.3 Progressive arterial desaturation

Participants were connected to the IHS throughout the exercise trial. During the 5 min lead‐in period, the inspiratory reservoir of the IHS was supplied with 21% O_2_ so that participants could establish a level of voluntary force generation that they perceived corresponded to the target intensity. At the start of MVC_50%_, the F_I_O_2_ of the inspirate was continuously reduced according to another second order polynomial function (Equation #2). For each experimental condition, a different function was developed to decrease S_P_O_2_ linearly over the target duration of the trial (Equation #2), according to the P_I_O_2_‐S_P_O_2_ relationship (Equation #1) previously established. (Equation #2) a–c exemplifies these functions for one subject:

(2a; FAST) $$P_{I}O_{2}=0.8x^{2}-16.4x+130.8,$$

(2b; MED) $$P_{I}O_{2}=0.08x^{2}-5.5x+130.8,$$

(2c; SLOW) $$P_{I}O_{2}=0.03x^{2}-3.3x+130.8$$

where

$$x=\text{Time}\;\left(\min\right).$$

### 2.4 Data acquisition and processing

All data were synchronously recorded (Power lab 16/35, ADInstruments, Australia), digitized and stored on a laptop computer for later analysis.

#### 2.4.1 Arterial oxygen saturation and heart rate

S_p_O_2_ and heart rate (HR) were sampled at 1 Hz using a pulse oximeter (Model # 7500, Nonin Medical, USA). The left earlobe was prepared by applying hyperemic cream (Finalgon, Boehringer‐Ingelheim, France) for 8 min. The probe was attached to the earlobe after cleaning the area with isopropyl alcohol. A 5 s sample was calculated just prior to each contraction.

#### 2.4.2 Dynamometry

Isometric knee extension force was measured by a custom built dynamometer. Participants were seated in the chair (Model # 41663, Quantum) with their hip and knee joints at 90°. A force transducer (SM‐500‐19, Interface, USA) was fixed to the chair and connected to a noncompliant cuff attached to the subject superior to the malleoli. Force signals were sampled at 1000 Hz and smoothed by calculating the moving average for every 35 points. Participants were instructed to hold their arms crossed across their chest and were not provided with any visual or verbal feedback about their force generation during contractions. During each MVC, participants were instructed to produce maximal force as quickly as possible while the investigators provided strong verbal encouragement. For MVC_PRE_, MVC_POST_, and MVC_REC_, the trial with the highest peak force was chosen for analysis. During MVC_50%_, force values were averaged over the middle 1 s segment of each contraction.

#### 2.4.3 Surface electromyography

Surface electromyography (sEMG) signals from the vastus medialis (VM) and vastus lateralis (VL) were measured via bipolar Ag‐Ag‐Cl surface electrodes (inter‐electrode distance 2 cm). Signals were amplified with an isolated differential amplifier (Dual Bioamp FE 135, ADInstruments, Australia), band pass filtered (10–500 Hz), and digitized with a sampling frequency of 2000 Hz. Electrodes were positioned over the muscle belly along the assumed angle of pennation after shaving and cleaning the area with isopropyl alcohol. Electrodes and wires were anchored using adhesive tape (Ref # 71443–02, Hypafix, Germany) to reduce movement artifact. sEMG signals were quantified by Root‐Mean‐Square (sEMG_RMS_). During MVC_50%_, sEMG_RMS_ values were averaged over the middle 1 s segment of each contraction and were normalized to the average of the first two contractions when S_P_O_2_ was >98%.

#### 2.4.4 Near infrared spectroscopy

Indicators of cerebral and muscle oxygenation were measured with near infra‐red spectroscopy (NIRS) (Niro 300, Hamamatsu, Japan) with a sampling rate of 1 Hz. NIRS continuously measured tissue oxygenation index (TOI) and oxyhemoglobin (O_2_Hb) in response to arterial desaturation. The cerebral probe was positioned over the left pre‐frontal cortex (PFC) 1 cm above the eyebrow and 1 cm to the left of the skull centre (Neary et al., 2008). The muscle probe was positioned over the right VL along the vertical axis of the thigh, approximately 10–14 cm from the knee joint (Bhambhani et al., 2001). The probes were fixed using a dense rubber vinyl holder and held in place with adhesive tape (Ref # 71443–02, Hypafix, Germany). TOI was calculated by the NIRS device as the ratio of oxygenated to total tissue hemoglobin. O_2_Hb values are expressed as relative changes from the first two contractions of MVC_50%_ trial, when S_P_O_2_ was >98%. NIRS parameters were averaged over the 5 s period just prior to the contraction.

#### 2.4.5 Ventilation

Minute ventilation (V̇_E_) was determined on a breath‐by‐breath basis using a heated pneumotach (Model # 3813, Hans Rudolph, USA) positioned on the expiratory limb of the sequential gas delivery breathing manifold. Airflow was sampled at 100 Hz and signals were low pass filtered (1 Hz). V̇_E_ was calculated as:

$${\dot{V}}_{E}=\frac{V_{T}}{\left(\frac{T_{B}}{60}\right)},$$

$$\begin{array}{c} V_{T}=\text{Tidal Volume}\;\left(L\right)=\text{integral of flow}-\text{time signal} \end{array}$$

where

$$\begin{array}{c} T_{B}=\text{Breathing Time}\;\left(s\right)=\text{time between the start of}\;\\ \;\mathrm{two}\;\text{succsessive expirations} \end{array}$$

Breath‐by‐breath V̇_E_ values were averaged over the 15 s period just prior to the contraction.

The magnitude of the ventilatory response (∆V̇_E_) during the four experimental conditions was calculated as the difference in V̇_E_ from the beginning to the end of the trial. In addition, the relationship between V̇_E_ and S_p_O_2_, the HYP ventilatory response (HVR), for the three HYP conditions was determined by dividing ∆V̇_E_ by the corresponding difference in S_p_O_2_ from baseline (BL) to maximal hypoxemia (MH) using the following equation (Goldberg et al., 2017):

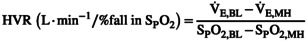




#### 2.4.6 Partial pressure of O_2_ and CO_2_

The partial pressure of O_2_ and carbon dioxide (CO_2_) were continuously measured at 100 Hz using a gas analyzer (O2CapB, Oxigraf, USA) sampling near the mouth. Inspired and end‐tidal values for O_2_ and CO_2_ were determined on a breath‐by‐breath basis using the built‐in functions of our data acquisition software (LabChart V8.0 Pro, ADInstruments, Australia). Inspired and end‐tidal values for O_2_ and CO_2_ were averaged over the 15 s period just prior to the contraction.

#### 2.4.7 Oxygen consumption

Oxygen consumption (V̇O_2_) was calculated breath‐by‐breath and averaged over the 15 s period just prior to each contraction. V̇O_2_ was calculated with the following equation (Slessarev et al., 2007):

$$\begin{array}{c} \dot{\dot{V}}O_{2}={\dot{V}}_{A}\times \left(F_{I}O_{2}-F_{A}O_{2}\right),\text{where} \end{array}$$

V̇_A_ = Alveolar ventilation (assumed to equal the flow rate of fresh gas into inspiratory reservoir of IHS).

F_A_O_2_ = Alveolar fractional O_2_ concentration (assumed to equal end‐tidal fractional O_2_ concentration).

#### 2.4.8 Calculations

The slope of the S_P_O_2_ versus time relationship was calculated using linear regression. To illustrate the change in the variables over the HYP trials, values were averaged over two contractions at defined S_P_O_2_ values (~100%, 90%, 80%, and 70%). During CON, variables were calculated at defined time points (0 min, 8.3 min, 16.7 min, and 25 min, which correspond to the start, one‐third, two‐thirds, and the end of the time required to complete the trial). For each variable, the data at these time points during CON were compared to the data collected at the defined S_P_O_2_ values of 100%, 90%, 80%, and 70% during each HYP trial respectively.

### 2.5 Statistical analysis

Results are presented as mean values ± standard deviation. Differences were considered significant when *p* < 0.05. The within‐subject standard deviation was estimated for CON throughout MVC_50%_ as the square root of the average variance across all eight participants to illustrate the variability of this exercise model. A one‐sample *t*‐test was conducted to determine if a difference existed between the self‐selected force voluntarily generated at the start of the exercise protocol and the target value of 50% MVC. A one‐way repeated measures analysis of variance was performed to evaluate the differences in the slope of the S_P_O_2_ versus time relationship between the experimental conditions. All other variables were analyzed using a two‐way repeated measures analysis of variance. When the assumption of sphericity was violated, a Greenhouse–Geisser correction was used. Significant main effects were followed up with pairwise comparisons and significant interaction effects were followed up with paired *t*‐tests. The *p*‐values reported for all follow up test were adjusted using a Bonferroni correction. All statistics were calculated using SPSS software (Version 26 for Mac, IBM).

## 3 RESULTS

### 3.1 Arterial saturation

**FIGURE 2:**
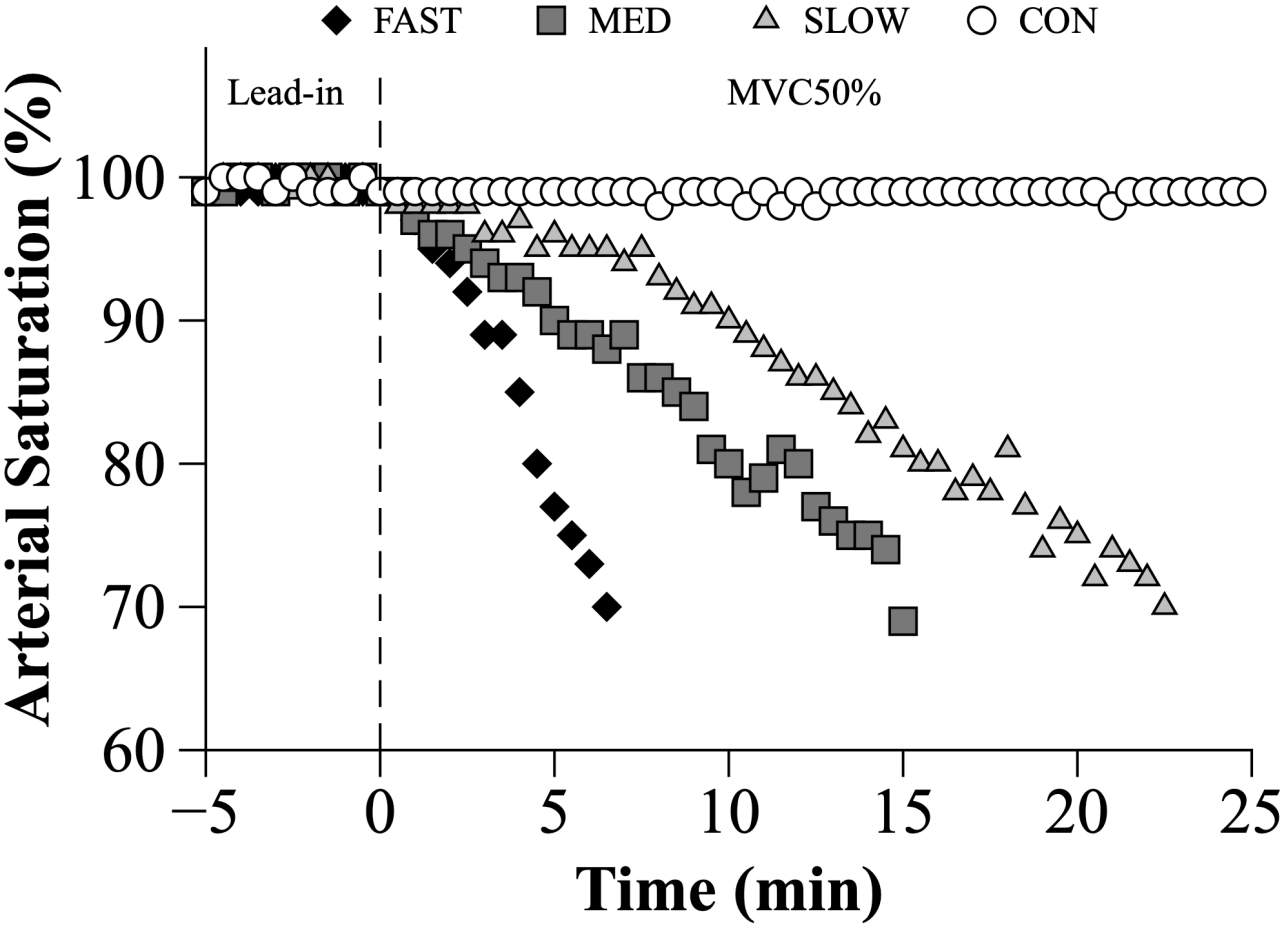
Representative data from one subject illustrating changes in arterial oxygen saturation over time. Time period between −5 and 0 min represents the lead‐in period in which the subject was provided 21% O_2_ so they could establish a level of voluntary effort that they perceived to correspond to 50% MVC force. MVC50% was initiated at 0 min when the desaturation protocol began.

S_P_O_2_ was at or above 98% at the start of each exercise trial. All participants completed the full desaturation procedure, where S_P_O_2_ decreased from baseline values to 70% in 5.3 ± 1.3 min, 11.8 ± 2.7 min, 19.9 ± 3.7 min for the FAST, MED, and SLOW conditions respectively. S_P_O_2_ remained at or above 99% throughout CON, which lasted 25.0 ± 0.0 min. The rate of arterial desaturation for FAST (−5.4 ± 1.0%•min^−1^), MED (−2.2 ± 0.5%•min^−1^), and SLOW (−1.4 ± 0.2%•min^−1^) was significantly different between the conditions (*p* < 0.001). Representative data from one subject, illustrating the S_P_O_2_ vs time relationship are shown in Figure 2.

### 3.2 Voluntary force production

Each participant maintained a consistent level of force generation in CON throughout MVC_50%_, with an overall within‐subject standard deviation of 5% MVC (64 N). Individual data are shown in Table 2.

**TABLE 2:** Variability in self‐selected force generated at a perceived intensity of 50% MVC during CON for each participant.

Subject	Mean force		Within subject SD		95% CI	
(#)	(% MVC)	(N)	(% MVC)	(N)	(% MVC)	(N)
1	34	270	4	28	33–35	262–277
2	59	386	5	35	58–61	376–396
3	46	375	6	45	45–48	363–388
4	41	553	6	75	39–42	531–574
5	47	447	5	47	46–48	434–460
6	43	700	4	69	42–44	681–719
7	57	1248	5	108	56–59	1217–1278
8	51	633	5	66	49–52	615–652

*Note*: For each participant, mean force, within‐subject standard deviation (SD), and 95% confidence interval (CI) were calculated using 51 contractions.

Abbreviations: MVC, maximal voluntary contraction; N, newtons.

**FIGURE 3:**
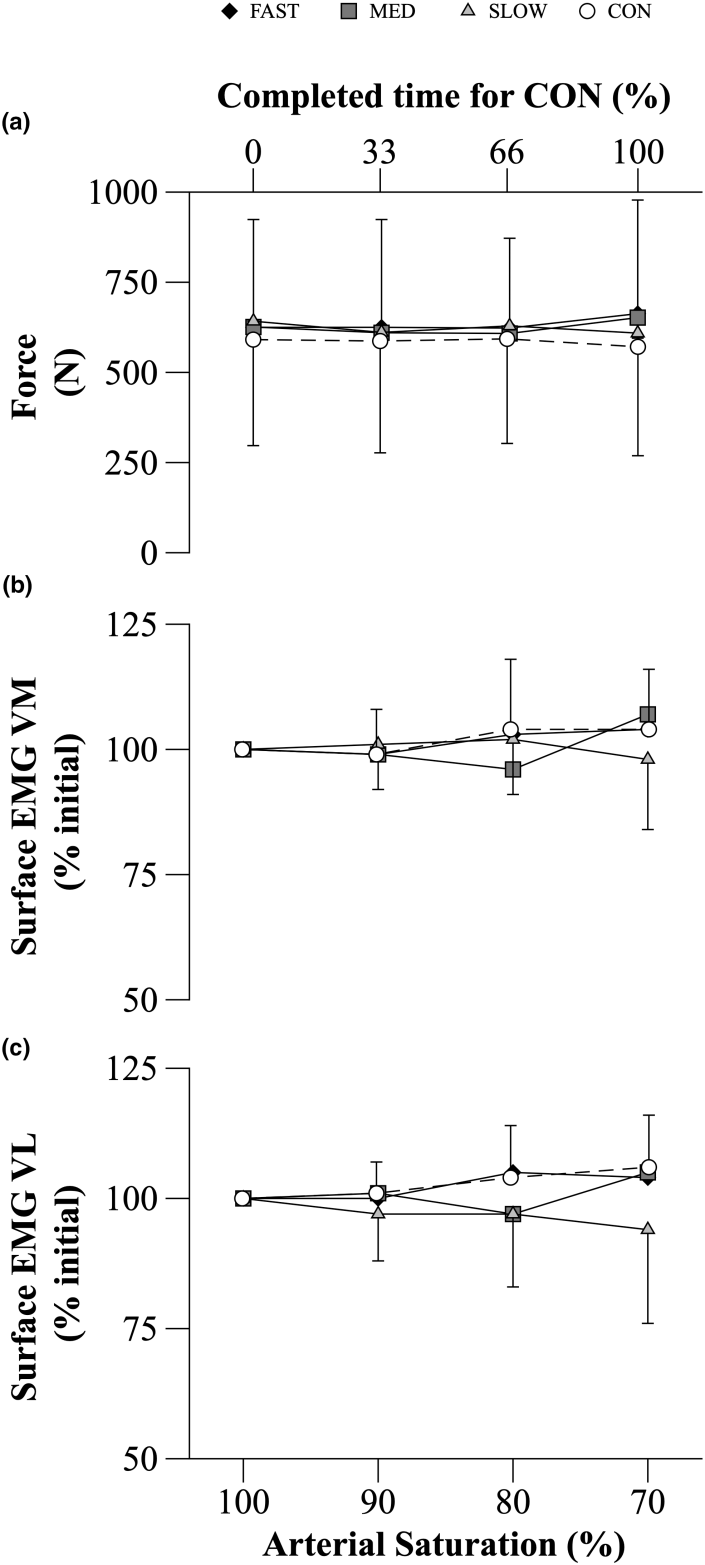
Force and surface EMG activity for vastus medialis (VM) and vastus lateralis (VL) throughout MVC_50%_ during HYP and CON trials. During CON, variables were calculated at the beginning, one‐third, two‐thirds, and at the end of the time required to complete the trial. These values were compared to the data collected at the defined S_P_O_2_ values of 100%, 90%, 80%, and 70% during each HYP trial respectively. Data are mean ± SD (*n* = 8). Data analyzed using a two‐way repeated measures ANOVA. No statistical differences were found.

No main (COND, *p* = 0.495; S_P_O_2_, *p* = 0.495) or interaction (COND x S_P_O_2_; *p* = 0.106) effects were detected for the force voluntarily generated during MVC_50%_. Force production at the start of MVC_50%_ was similar across trials. As the HYP and CON trials progressed, self‐selected force generation remained at a similar level throughout the exercise protocol (Figure 3). The self‐selected force generated at the start of MVC_50%_ was not statistically different than the target intensity of 50% MVC for FAST (50 ± 6% MVC, t(7) = 0.057, *p* = 0.956), MED (48 ± 7% MVC, t(7) = −0.664, *p* = 0.528), SLOW (51 ± 7% MVC, t(7) = 0.308, *p* = 0.767), and CON (49 ± 7% MVC, t(7) = −0.568, *p* = 0.588).

**FIGURE 4:**
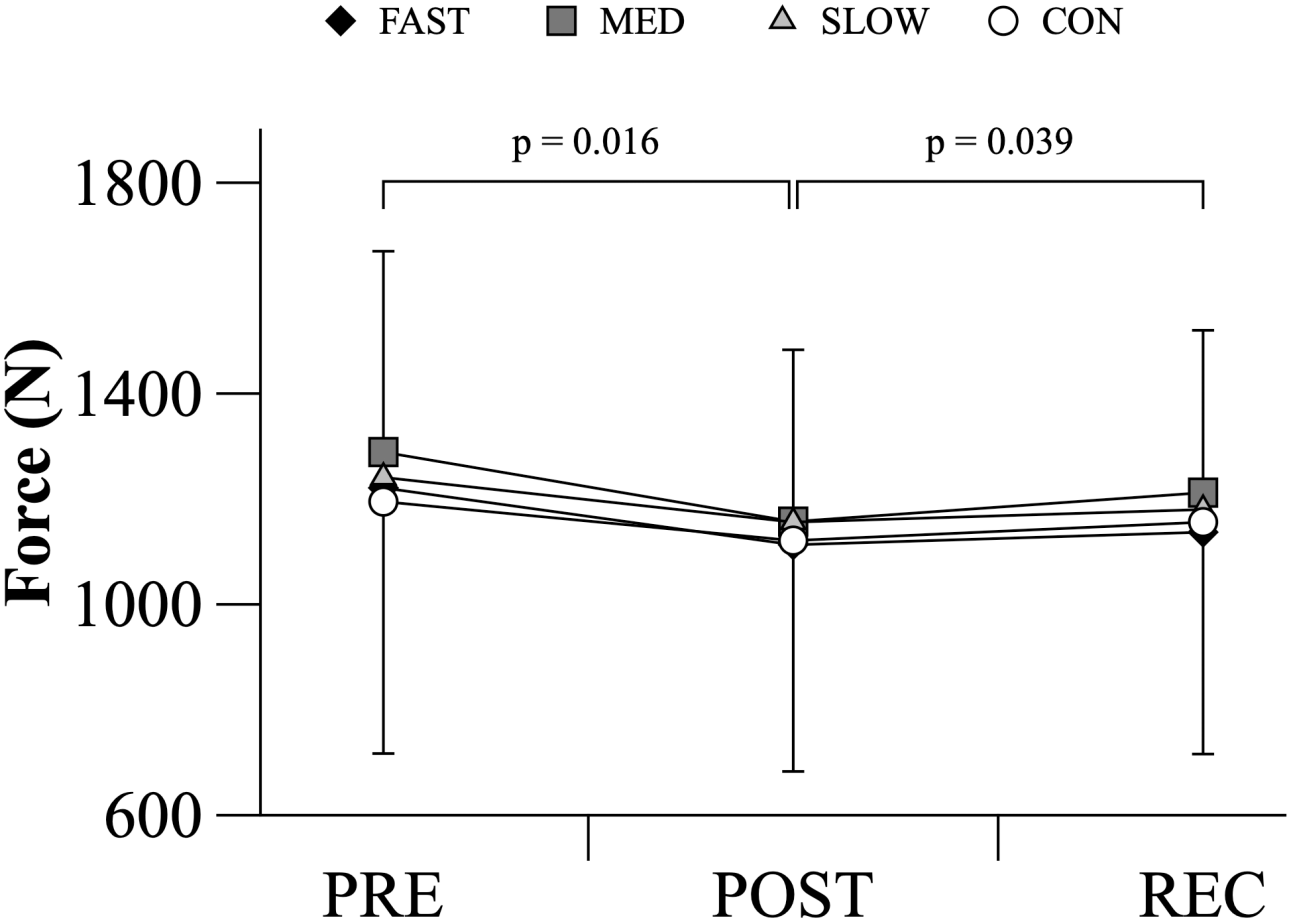
Maximal voluntary contraction force generated throughout the exercise protocol during HYP and CON trials. During CON, variables were calculated at the beginning, one‐third, two‐thirds, and at the end of the time required to complete the trial. These values were compared to the data collected at the defined S_P_O_2_ values of 100%, 90%, 80%, and 70% during each HYP trial respectively. Data are mean ± SD (*n* = 8). Data analyzed using a two‐way repeated measures ANOVA. Significant interaction effects were followed up with pairwise comparisons. The p‐values reported were adjusted using a Bonferroni correction. Statistical significance was accepted at *p* < 0.05.

There was a main effect of TIME on MVC (*p* = 0.002). MVC_PRE_ was similar for all trials. Upon completion of MVC_50%_, MVC_POST_ decreased across all conditions to a similar level. After the recovery period, MVC_REC_ values significantly increased to levels that were not different than MVC_PRE_. (Figure 4).

### 3.3 Surface electromyography

No main (*p* > 0.05) or interaction (*p* > 0.05) effects were detected for sEMG_RMS_ during MVC_50%_ of either VM or VL. sEMG_RMS_ of both muscles remained at a similar level throughout MVC_50%_ across all conditions (Figure 3).

### 3.4 Near infrared spectroscopy

**FIGURE 5:**
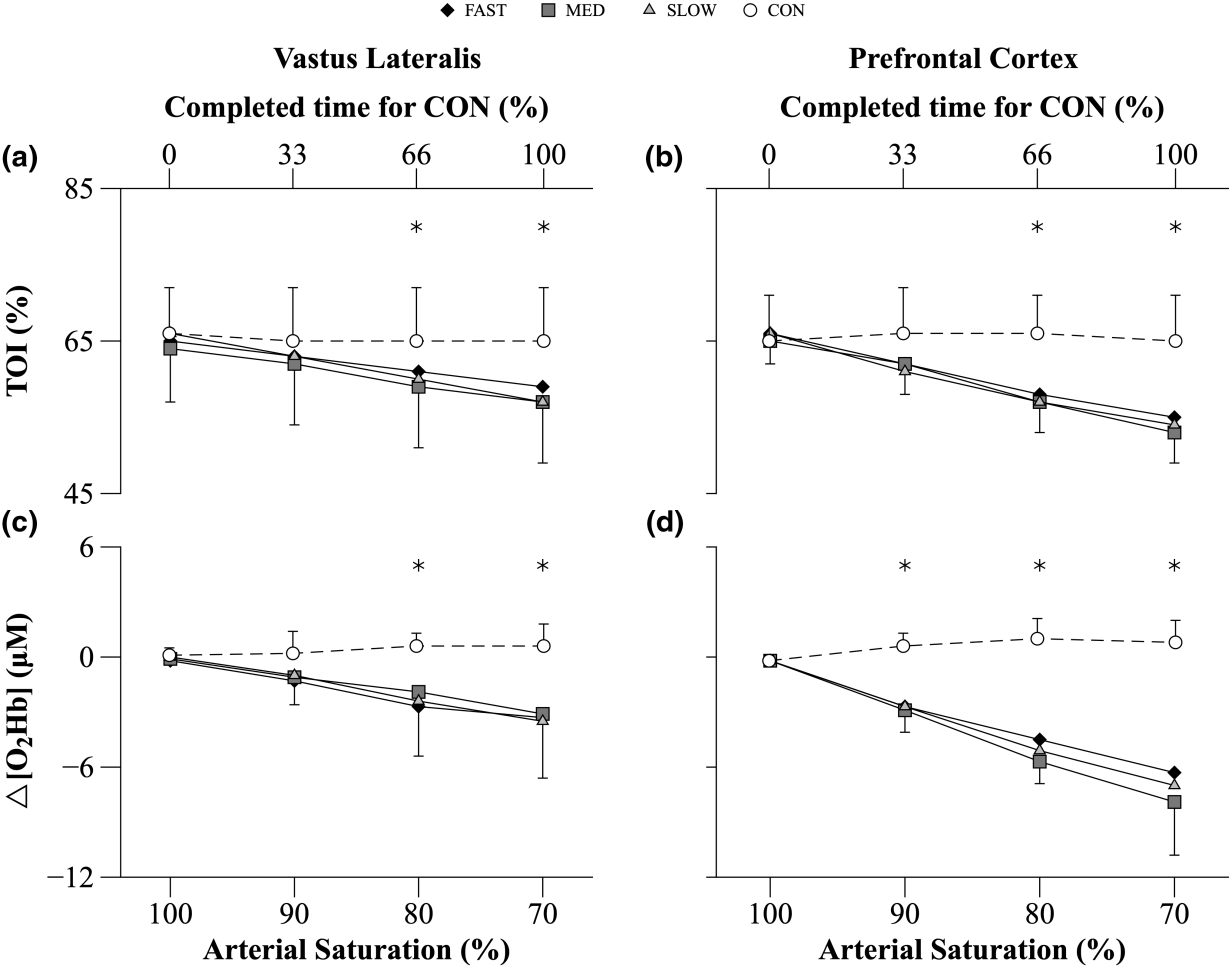
Tissue oxygenation index (TOI) and ∆ Oxyhemoglobin (O_2_Hb) for vastus lateralis and prefrontal cortex throughout the exercise protocol during HYP and CON trials. During CON, variables were calculated at the beginning, one‐third, two‐thirds, and at the end of the time required to complete the trial. These values were compared to the data collected at the defined S_P_O_2_ values of 100%, 90%, 80%, and 70% during each HYP trial respectively. Data are mean ± SD (*n* = 8). Data analyzed using a two‐way repeated measures ANOVA. Significant interaction effects were followed up with paired *t*‐tests. The *p*‐values reported were adjusted using a Bonferroni correction. *FAST, MED, and SLOW significantly different from CON (*p* < 0.05).

There was an interaction effect between COND and S_P_O_2_ (*p* < 0.001) on TOI for both the VL and PFC. Follow up tests revealed that the baseline TOI values were similar across all conditions for each tissue. TOI did not significantly change from baseline throughout the CON trial. With reductions in S_P_O_2_, the decreases in TOI during FAST, MED, and SLOW were similar and all were less than CON when S_P_O_2_ was at 80% (*p* ≤ 0.048) and 70% (*p* ≤ 0.042) in both sampling locations (Figure 5).

There was an interaction effect between COND and S_P_O_2_ (*p* = 0.001) on O_2_Hb for both VL and PFC. Follow up tests revealed that baseline O_2_Hb values were similar across all conditions for each tissue. O_2_Hb remained at baseline levels throughout the CON trial for both tissues. With the decline of S_P_O_2_, O_2_Hb decreased during FAST, MED, and SLOW to a similar level and all were less than CON when S_P_O_2_ reached 80% in the VL (*p* ≤ 0.03) and 90% in the PFC (*p* ≤ 0.006) (Figure 5).

### 3.5 Heart rate

**FIGURE 6:**
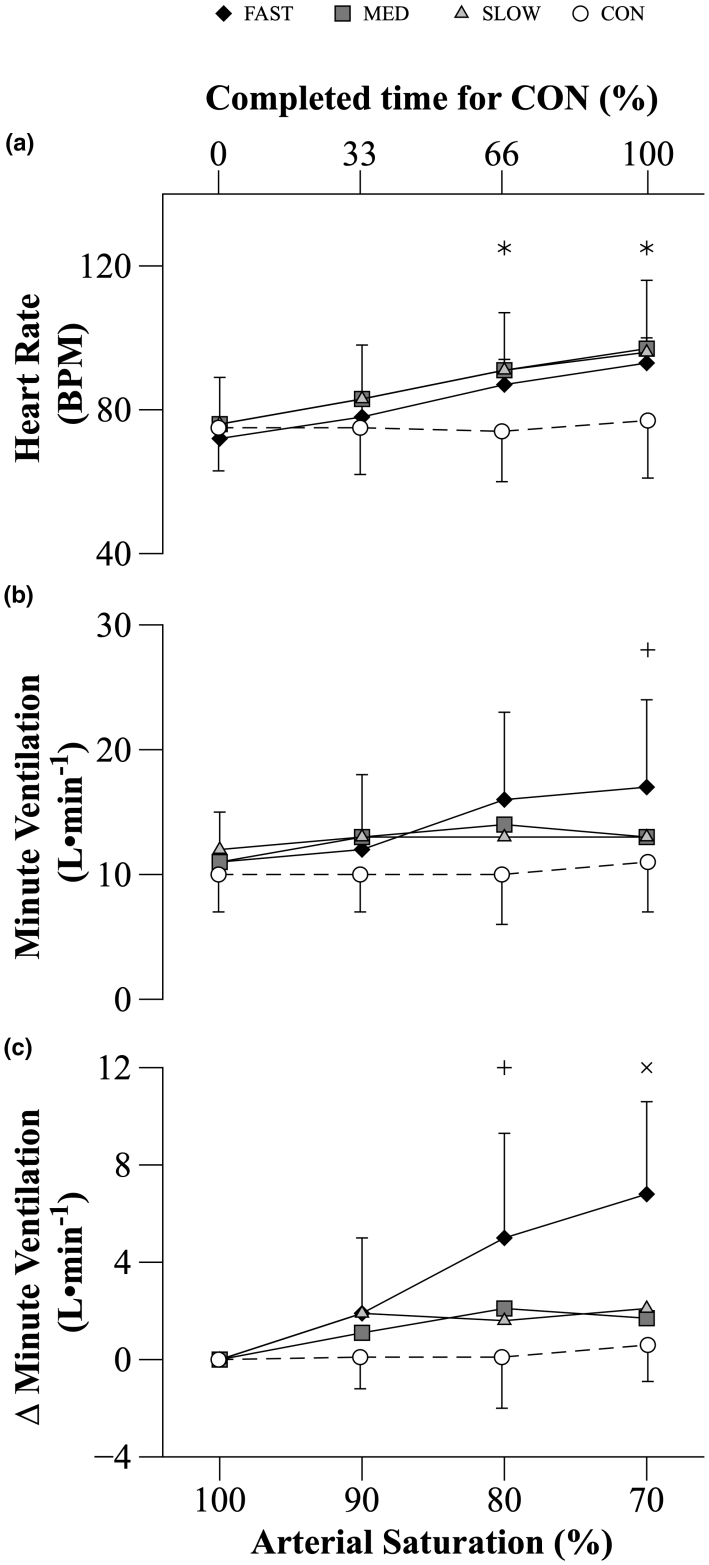
Heart rate, Minute ventilation, and ∆ Minute ventilation throughout MVC_50%_ during HYP and CON trials. During CON, variables were calculated at the beginning, one‐third, two‐thirds, and at the end of the time required to complete the trial. These values were compared to the data collected at the defined S_P_O_2_ values of 100%, 90%, 80%, and 70% during each HYP trial respectively. Data are mean ± SD (*n* = 8). Data analyzed using a two‐way repeated measures ANOVA. Significant interaction effects were followed up with paired t‐tests. The p‐values reported were adjusted using a Bonferroni correction. * FAST, MED, and SLOW significantly different from CON (*p* < 0.05). ^+^ FAST significantly different from CON (*p* < 0.05). ^×^ FAST significantly different from MED, SLOW, CON (*p* < 0.05).

There was an interaction effect between COND and S_P_O_2_ (*p* < 0.001) on HR. Baseline HR values were similar across all conditions. HR did not significantly change from baseline levels throughout the CON trial. With reductions in S_P_O_2_, the increase in HR was similar for FAST, MED, and SLOW and all were greater than CON when S_P_O_2_ was at 80% (*p* ≤ 0.024) and 70% (*p* ≤ 0.03) (Figure 6).

### 3.6 Ventilation

There was an interaction effect between COND and S_P_O_2_ on V̇_E_ (*p* = 0.01). The baseline V̇_E_ was not different among the four experimental conditions. V̇_E_ did not significantly change from baseline levels throughout the CON trial (Figure 6b). With reductions in S_P_O_2_, ∆V̇_E_ during MED and SLOW were similar, and both were less than FAST when S_P_O_2_ reached 70% (*p* ≤ 0.048; Figure 6c). The HVR, expressed as the increase of ventilation to the percentage fall in S_P_O_2_, was −0.28 ± 0.17 L•min^−1^ / % fall of S_P_O_2_ (FAST), −0.07 ± 0.11 L•min^−1^ / % fall of S_P_O_2_ (MED), and − 0.07 ± 0.09 L•min^−1^ / % fall of S_P_O_2_ (SLOW). The HVR during FAST was significantly higher than that in MED (*p* ≤ 0.026) and SLOW (*p* ≤ 0.048).

### 3.7 Oxygen consumption

**FIGURE 7:**
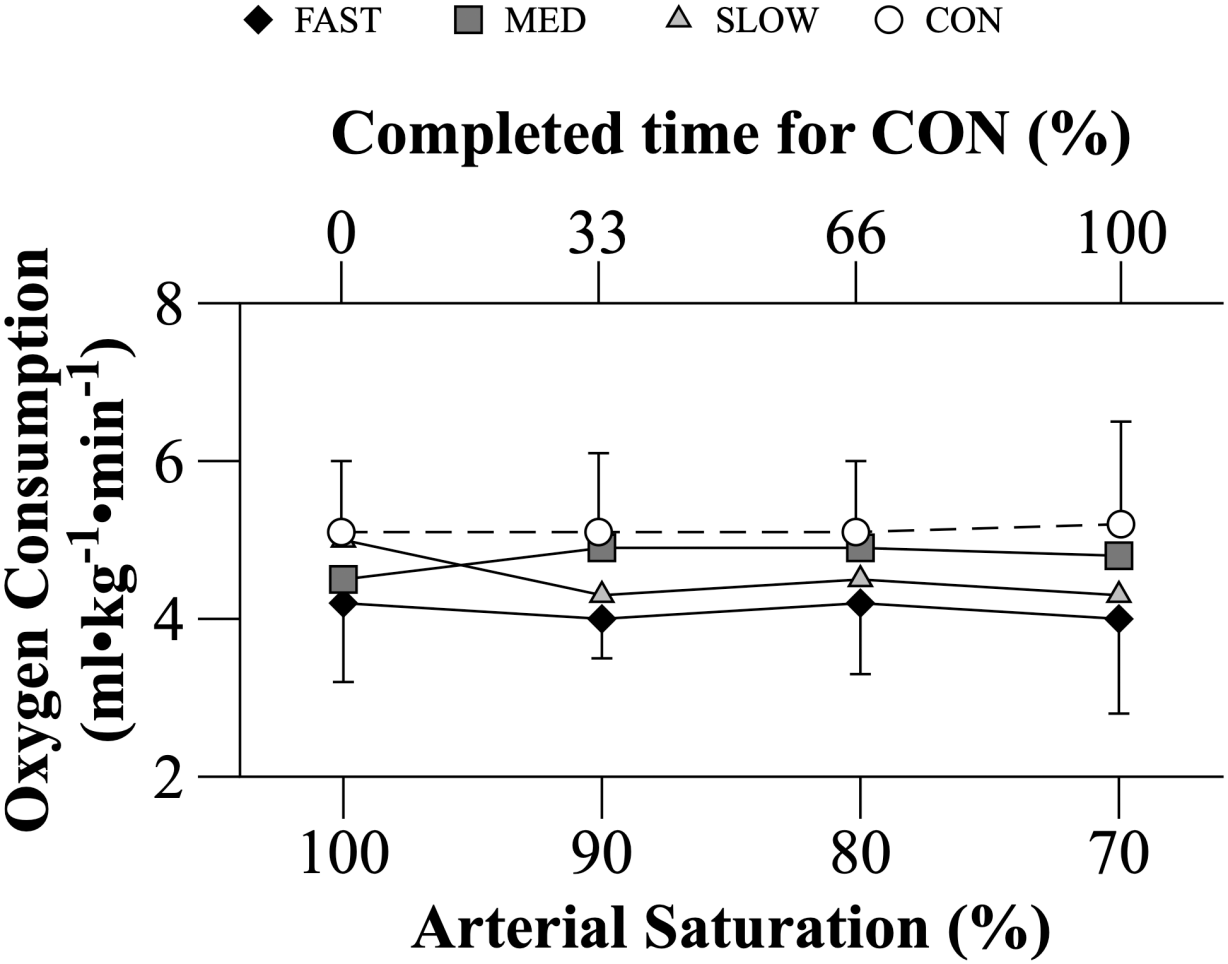
Oxygen consumption throughout MVC_50%_ during HYP and CON trials. During CON, variables were calculated at the beginning, one‐third, two‐thirds, and at the end of the time required to complete the trial. These values were compared to the data collected at the defined S_P_O_2_ values of 100%, 90%, 80%, and 70% during each HYP trial respectively. Data are mean ± SD (*n* = 8). Data analyzed using a two‐way repeated measures ANOVA. No statistical differences were found.

No main (COND, *p* = 0.105; S_P_O_2_, *p* = 0.655) or interaction (COND x S_P_O_2_; *p* = 0.688) effects were detected for VO_2_ during MVC_50%_. V̇O_2_ at the start of MVC_50%_ was similar across all trials and remained at that level as exercise progressed (Figure 7).

## 4 DISCUSSION

We have previously demonstrated that arterial desaturation is associated with a reduction in self‐selected exercise intensity corresponding to a given RPE during cycling exercise, and that this reduction is magnified with faster rates of arterial desaturation (Farra et al., 2017). The aim of the current study was to examine potential mechanisms that regulate this rate dependent relationship. Our approach involved repeated 3 s unilateral, intermittent, isometric knee extensions with a 27 s recovery period between contractions. The goal of the relatively long recovery period was to attenuate accumulation of metabolic by‐products associated with impaired muscle contractility and also with stimulation of peripheral muscle afferents. We proposed that by reducing muscular metabolic strain in this fashion, the effect of inhibitory sensory feedback would be minimized, while accentuating any direct influence of arterial desaturation rate on self‐selected force production. The main finding of this study is that a decrease in absolute O_2_ availability and faster rates of arterial desaturation both did not alter self‐selected force production during intermittent, unilateral, isometric knee extension exercise at a fixed level of perceptual effort, even when intramuscular metabolic strain within the knee extensors was presumably reduced. However, the magnitude of the ventilatory response to progressive isocapnic hypoxia was influenced by the rate of decrease in S_P_O_2_. These findings suggest that while the rate of arterial desaturation directly influences certain physiological responses, muscle activation as assessed in this study is not among them.

This investigation corroborates previous research demonstrating that the HVR to isocapnic progressive hypoxia is higher when arterial desaturation is induced at a faster rate (Igarashi et al., 1995). Several mechanisms may explain the difference in the HVR between FAST and the remaining experimental conditions in this investigation. First, sensory receptors display changes in sensitivity in response to stimuli over time (Webster, 2012). Some studies have demonstrated a gradual and significant decline in peripheral chemoreceptor firing after reaching a maximum discharge rate during constant isocapnic hypoxia (Khamnei & Robbins, 1990; Li et al., 1990), while others have not (Barnard et al., 1987). Reduced levels of peripheral chemoreceptor feedback projecting onto supraspinal centers would decrease the excitatory synaptic drive of respiratory neurons. Consequently, should the peripheral chemoreceptors adapt over time, the longer it takes to reach a specific S_P_O_2_, the lower their activation and associated ventilatory response will be. Second, depression of neuronal excitability resulting from the local accumulation of inhibitory neurotransmitters and/or neuromodulators in response to prolonged hypoxia may lower respiratory drive. As the hypoxia‐induced alterations of these neuroeffectors occur over time (Richter et al., 1999), the likelihood of neuronal hyperpolarization increases with longer hypoxia exposure. Of these neuromodulators, increases in adenosine may be a candidate contributing to the depression of the HVR with slower arterial desaturation rates, as Igarashi et al. (1995) demonstrated that the time dependency of the HVR disappeared following a pretreatment with theophylline, an adenosine antagonist. Interestingly, while the HVR in this investigation was dependent on the rate of arterial desaturation, the HVR during whole‐body cycling exercise was not (Farra et al., 2017). This discrepancy suggests that the additional stimuli that contribute to the control of ventilation during whole‐body exercise (e.g., central command, metaboreflex, mechanoreflex, body temperature) override the mechanisms that suppress the HVR during slower arterial desaturation rates.

When exercising in HYP conditions, feedback from the peripheral chemoreceptors, central chemoreceptors, and group III/IV muscle afferents play an essential role in maintaining a respiratory steady state. Collectively, these sensors project onto and alter the output of the solitary tract nucleus (NTS). NTS projects onto several targets including the insular cortex (Craig, 2003). Williamson et al. (2006) describe the insular cortex as a likely area involved in the generation of perceived exertion, while Tanaka and Watanabe (2012) describe the insular cortex as an area that projects inhibitory signals onto the motor cortex. If we consider these models collectively, we may speculate that the afferent feedback mediated change in NTS output influences V̇_E_ (Mateika & Duffin, 1995), as well as CMD descending from the motor cortex (Amann et al., 2006; Amann et al., 2008; Amann et al., 2009; Amann et al., 2013).

Although this investigation demonstrated that arterial desaturation rate directly influenced ventilatory responses, a concomitant effect on muscle activation was not observed. This investigation was conducted in an isocapnic background, so it is reasonable to speculate that central chemoreceptor discharge (Mateika & Duffin, 1995) and the effect of CO_2_ on cerebral blood flow (Battisti‐Charbonney et al., 2011) were likely controlled during the exercise protocol. Accordingly, Farra et al. (2017) speculated that the rate dependent relationship between arterial desaturation and self‐selected exercise intensity may be regulated by feedback arising from either the peripheral chemoreceptors and/or peripheral muscle afferents. The current investigation demonstrated that faster rates of arterial desaturation did not alter self‐selected force generation when the intramuscular metabolic disturbance was presumably relatively trivial. This finding does not support the hypothesis that peripheral chemoreceptor feedback arising during isocapnic progressive hypoxia contributes to the regulation of self‐selected exercise intensity.

The results of the current investigation contrast with previous research that reported an exacerbated decline in voluntary force production during intermittent isometric contractions in acute hypoxia (Fulco et al., 1994; Fulco et al., 2001; Katayama et al., 2007; Katayama et al., 2010; Rupp et al., 2015). Although the level of hypoxemia and intensity of voluntary force production were similar to the current study, previous research used relatively short rest periods (3–10 s) between contractions that resulted in the development of peripheral (Romer et al., 2007) and central (Millet et al., 2009) fatigue that were likely independent of hypoxia. These reductions in voluntary force production may have been the consequence of increasing concentrations of inhibitory metabolic by‐products (Allen et al., 2008; Rotto & Kaufman, 1988). However, the restorative effect of the longer rest period in this investigation presumably maintained the muscular metabolic milieu closer to pre‐exercise conditions. A key feature of the current study was that the exercise protocol reduced metabolic perturbation, and associated inhibitory peripheral muscle afferent feedback, within the contracting muscles. This protocol facilitated testing whether the rate of arterial desaturation directly influences muscle activation independently from the magnitude of desaturation.

Our observations agree with reports in which MVC force production decreased after submaximal exercise of an isolated muscle (Fulco et al., 1994; Fulco et al., 2001; Katayama et al., 2007), and suggests the development of fatigue during MVC_POST_. Our results also demonstrated that hypoxia did not exacerbate the impairment incurred by exercise, as MVC_POST_ in the HYP and CON trials were similar. However, it is important to acknowledge the limitations of the MVC_POST_ measurement before drawing conclusions from that data. When assessing MVC force, participants are generally given multiple attempts at producing maximal force output and are provided with visual feedback (Allen et al., 1995; Garland, 1991; Garland & McComas, 1990; Goodall et al., 2010; Grisdale et al., 1990; Kalmar & Cafarelli, 2006; Millet et al., 2012). While most participants can achieve maximal muscle force generation voluntarily, they only do so in approximately 25% of attempted MVCs (Allen et al., 1995). When MVC force is based on a single attempt, as was the case with MVC_POST_, the validity of that trial as “maximal” cannot be confirmed. Nevertheless, the purpose of this study was to assess the influence of different rates of arterial desaturation on self‐selected force production. This prevented multiple MVC attempts following the MVC_50%_ trial, as it would require holding S_P_O_2_ at 70% for several minutes, forcing the instantaneous arterial desaturation rate to be equal to zero. When the instantaneous arterial desaturation rate is zero, assessment of the “rate of change” effect is no longer possible. Furthermore, the provision of visual feedback has been shown to be very important for maximal force generation, as this type of informative feedback may improve motivational encouragement (Peacock et al., 1981). However, asking participants to generate a force that they perceived to be equivalent to 50% MVC was a vital component of this study, which precluded the use of visual feedback. Therefore, the MVC_POST_ data presented in this study may not truly reflect the force generating capabilities of participants after exercise in both the HYP and CON trials.

### 4.1 Limitations

The level of intramuscular metabolic strain was not directly assessed to confirm its stability over the trials with reductions in S_P_O_2_. However, previous research has demonstrated that muscle blood flow and O_2_ extraction were preserved when exposed to hypoxia (inspired O_2_ = 14%) at rest, as reductions in arterial O_2_ content were matched by concomitant reductions in venous O_2_ content (Heinonen et al., 2010). As participants in this study recovered for 27 s between each 3 s contraction, the knee extensor muscles were in a resting state for approximately 89% of the exercise protocol. In combination with the submaximal nature of the contractions, the O_2_ requirement of muscles was likely adequately met and did not lead to significant metabolic strain.

The possibility that group III/IV muscle afferent output changed with hypoxia cannot be excluded, as the baseline discharge of these afferents increases during HYP exposure independent of muscular metabolic changes (Hill et al., 1992). Hill et al. (1992) demonstrated that the hypoxia‐induced changes in afferent output were small, suggesting that muscle hypoxia has a minor influence on these receptors. However, given their prolific innervation of muscle tissue, small changes in their individual discharge rates may substantially influence their collective input into the CNS (Gandevia, 1998).

## 5 CONCLUSIONS

Neither decreases in S_P_O_2_ nor the rate at which S_P_O_2_ was reduced altered self‐selected isometric force production at a perceived intensity of 50% MVC when the active muscle was allowed to recover between contractions. However, the ventilatory response to isocapnic progressive hypoxia was modified by the rate of arterial desaturation. Although certain physiological responses appear to be affected by the rate of HYP strain development, these findings do not support the hypothesis that muscle activation is directly and independently influenced by the rate of arterial desaturation. Our understanding of mechanisms that limit human exercise performance would be improved with further research about whether the rate of change in muscular metabolic strain, and accompanying peripheral muscle afferent feedback, has a role in regulating self‐selected exercise intensity.

## FUNDING INFORMATION

This work was supported by the Province of Ontario's Research Program in Applied Sport Science.

## CONFLICT OF INTEREST STATEMENT

The authors declare no conflicts of interest, financial or otherwise.

## ETHICS STATEMENT

The Health Sciences Research Ethics Board of the University of Toronto approved the protocol for this study (Protocol Reference # 31141). All experiments conformed to the standards set by the Declaration of Helsinki.

## Data Availability

All data generated or analyzed during this study are included in this published article.
